# Postgraduate family medicine training in Southeast Asia

**DOI:** 10.51866/cm.903

**Published:** 2025-05-01

**Authors:** Apichai Wattanapisit, Krishna Suvarnabhumi, Wichuda Jiraporncharoen, Indah Suci Widyahening, Oua Phimmasarn, Khamphanh Prabouasone, Ping Yein Lee, Adina Abdullah, Thin Nyein Nyein Aung, Myint Zaw, Leilani Apostol Nicodemus, Chirk Jenn Ng, Ho Thi Kim Thanh, Pham Le An

**Affiliations:** 1 MD, Dip. Thai Board of Family Medicine, Academic Fellowship (Family Medicine, Department of Clinical Medicine, School of Medicine, Walailak University, Nakhon Si Thammarat, Thailand.; 2 Family Medicine Clinic, Walailak University Hospital, Nakhon Si Thammarat, Thailand.; 3 The Excellent Center of Community Health Promotion, Walailak University, Nakhon Si Thammarat, Thailand. Email: apichai.wa@wu.ac.th; 4 MD, Dip. Thai Board of Family Medicine, Academic Fellowship (Family Medicine), MSc (Medical Education, Department of Family and Preventive Medicine, Faculty of Medicine, Prince of Songkla University, Songkhla, Thailand. Email: krishna.s@psu.ac.th; 5 MD, Dip. Thai Board of Family Medicine, MSc (Medical Education, Department of Family Medicine, Chiang Mai University, Chiang Mai, Thailand.; 6 MS, MSc.CM-FM, PhD, Department of Community Medicine, Faculty of Medicine, Universitas Indonesia, Jakarta, Indonesia.; 7 Primary Health Care Research and Innovation Center, Indonesia Medical Education and Research Institute (IMERI), Faculty of Medicine Universitas Indonesia, Jakarta, Indonesia.; 8 MD, FM, Neurologist, Community Medicine and Family Medicine, University of Health Sciences of Lao PDR, Vientiane, Laos.; 9 MD, DTM&H, MCTM, PhD, Faculty of Medicine, University of Health Sciences of Lao PDR, Vientiane, Laos.; 10 MBBS, MMed (Fam Med, UMeHealth Unit, Faculty of Medicine, Universiti Malaya, Kuala Lumpur, Malaysia.; 11 BMedSci (Hons), MBBS, MMed (Fam Med), PhD, Department of Primary Care Medicine, Faculty of Medicine, Universiti Malaya, Malaysia.; 12 MBBS, MMedSc (Rehabilitation Medicine), PhD, Department of Family Medicine, Faculty of Medicine, Chiang Mai University, Chiang Mai, Thailand.; 13 MBBS, Dip Med Sc (Family Medicine), Certified Fellowship of Myanmar Academy of Family Physician General Practitioner Society, Myanmar Medical Association Yangon, Myanmar.; 14 MD, MSc-FM, FPAFP, Department of Family and Community Medicine, University of the Philippines, Manila, Philippines.; 15 MBBS, MMed (Family Medicine), PhD Department of Research, SingHealth Polyclinics, Singapore.; 16 Health Services and Systems Research, Duke-NUS Medical School, Singapore.; 17 MD, Master Degree-PhD Internal Medicine, Family medicine Family Medicine Department, Hanoi Medical University, Hanoi, Vietnam.; 18 Family Medicine and Community Health Care Center, Hanoi Medical University Hospital, Hanoi, Vietnam.; 19 MD, PhD Pediatrician, Family Medicine, Family Medicine Department, Ho Chi Minh University of Pharmacy and Medicine, at Chi Minh city, Vietnam.

**Keywords:** Family practice, Graduate medical education, Medicine, Southeast Asia

## Abstract

Family medicine is a medical discipline that has been recognised globally for nearly six decades. Postgraduate family medicine training was introduced more recently in Southeast Asia. This article presents the characteristics of postgraduate family medicine training in eight Southeast Asian countries: Indonesia, Laos, Malaysia, Myanmar, the Philippines, Singapore, Thailand and Vietnam. The duration of training varies across countries and programmes within each country, ranging from 1 to 5 years. Completion of training leads to qualifications such as master’s degree, diploma or certificate. Some countries also offer further training following postgraduate family medicine training, classified into two types: capacity-based training (e.g. family medicine fellowship) and discipline-based training (e.g. palliative care fellowship). The increasing burden of non-communicable diseases and the ageing population as well as the shortage of family physicians are significant concerns and challenges that influence postgraduate family medicine training in Southeast Asia.

## Introduction

Family medicine is a relatively young discipline in medicine. Training in family medicine began in the 1960s in the United Kingdom, Canada and the United States.^1^ Family medicine residency programmes were established at the University of Western Ontario and the University of Calgary in Canada in 1966.^2^ In 1968, the College of General Practitioners (founded in 1954) was renamed the College of Family Physicians of Canada to align with the residency training.^2,3^ This marked a key milestone in the formalisation of family medicine training. Postgraduate family medicine training has expanded globally^4^ and influenced practices of primary care and primary healthcare in many countries.^1,2^ In addition, family medicine is key in strengthening universal health coverage.^5^

In Southeast Asia, which comprises 11 countries with a population of over 680 million,^6^ family medicine develops at a different pace across the region. Two key publications have highlighted the availability and characteristics of postgraduate family medicine training programmes in the Asia Pacific and globally.^4,7^ Postgraduate family medicine training programmes are commonly offered as family medicine residency programmes, master’s degrees or diplomas.^4^ In addition, the Wilfrid Laurier University’s website (https://globalfamilymedicine.org/) compiles information on global family medicine, including data from nine Southeast Asian countries.^8^ These resources provide a comprehensive understanding and valuable insights into postgraduate family medicine training in Southeast Asia. However, obtaining updated information from each country is challenging due to factors such as language barriers and limited access to available documents.

This article aims to explore the characteristics, challenges and future trends of postgraduate family medicine training in Southeast Asia. Lead authors from Thailand invited potential coauthors from Southeast Asian countries through academic networks and a snowball technique. The content of this article was finalised in January 2025 based on literature review and contributions by authors from each country.

**Postgraduate training in family medicine and special interests**

The current postgraduate training programmes in eight countries - Indonesia, Laos, Malaysia, Myanmar, the Philippines, Singapore, Thailand and Vietnam - are summarised in Table 1. Postgraduate family medicine training programmes are classified into four categories: clinical residency, master’s degree, diploma and certificate. These training programmes last 1-5 years. Some countries also offer additional programmes following postgraduate family medicine training, which fall into two main types: capacity-based training (e.g. community-oriented primary care and family-oriented medical care in Indonesia and family medicine fellowship in Singapore) and discipline-based training (e.g. palliative care, geriatric family medicine and addiction family medicine in Thailand; clinical fellowship programmes in hospice and palliative care, geriatrics, preventive and lifestyle medicine, toxicology, addiction medicine and medical nutrition in the Philippines; and non-communicable diseases [NCDs], communicable diseases, palliative medicine, mental health, addiction medicine, sexual and reproductive health, geriatrics, child health, adolescent health and rehabilitation in Malaysia).

**Table 1:** Postgraduate training in family medicine and special interests in Southeast Asia.

Country (year of the beginning of training)	Training regulator/institution	Programme title	Duration of training	Qualification	Further training (after family medicine training)
Indonesia (2017)^*^	**Regulator:** Indonesian College of Family Medicine Primary Care **Implementer:** - University-based institutions (regular programme) - College (recognition of prior competencies - RKL) - Hospital-based institutions (under development)	Residency	3.5 years/7 semesters (regular) 1-2 years (RKL)	Specialist degree	**Subspecialist training (2 years):** - Community-oriented primary care - Family-oriented medical care
Laos (2005)	**Regulator:** - Department of Community & Family Medicine, Faculty of Medicine **Implementer:** - Hospital- and community-based	Lao Family Medicine Specialist Programme	3 years	Specialist degree	None
Malaysia (1989)	Public universities: Universiti Kebangsaan Malaysia, Universiti Malaya, Universiti Sains Malaysia, Universiti Putra Malaysia, International Islamic University Malaysia and Universiti Teknologi MARA	Master of Family Medicine	4 years	Master’s degree	Non-communicable diseases, communicable diseases, palliative medicine, mental health, addiction medicine, sexual and reproductive health, geriatrics, child health, adolescent health and rehabilitation
	Academy of Family Physicians of Malaysia	Vocational Training Programme	2 years	Membership	
		Diploma	2 years (part-time)	Certificate	
Myanmar (2015)	Myanmar Academy of Family Physicians	Diploma in Family Medicine	1 year (47 weeks total)	Diploma	None
Philippines (1972)	Philippine Academy of Family Physicians (accreditation body) Philippine Regulation Commission (Professional Qualification Framework adherence)	Residency	3 years (hospital-based training) 4-5 years (practice-based training)	Specialist	- Hospice and palliative medicine (1 year) - Geriatrics (2 years) - Preventive and lifestyle medicine (2 years) - Toxicology (2 years) - Medical nutrition (2 years) - Addiction medicine (2 years)
	University of the Philippines Manila College of Medicine	Master in Clinical Medicine - Family Medicine	2 years of coursework and 2-3 years of thesis project	MSc.CM-FM	None
Singapore (1993)	Training provided by the respective public primary care institutions: National Healthcare Group Polyclinics National University Polyclinics SingHealth Polyclinics Examination conducted by: Division of Graduate Medical Studies National University of Singapore (NUS Medicine)	Family Medicine Residency Programme	3 years	Master of Medicine (Family Medicine)	Advanced Specialty Training in Family Medicine (2 years) leading to Fellowship (FCFPS) by Assessment Graduate Diploma Programmes: - Graduate Diploma in Palliative Care - Graduate Diploma in Geriatric Medicine - Graduate Diploma in Mental Health - Graduate Diploma in Child and Adolescent Health
	College of Family Physicians Singapore Examination conducted by: Division of Graduate Medical Studies National University of Singapore (NUS Medicine)	Master of Medicine (Family Medicine) College Programme	2 years (after completion of Graduate Diploma in Family Medicine)	Master of Medicine (Family Medicine)	
	College of Family Physicians Singapore Examination conducted by: Division of Graduate Medical Studies National University of Singapore (NUS Medicine)	Graduate Diploma in Family Medicine	2 years	Graduate Diploma in Family Medicine	
Thailand (1998)	Royal College of Family Physicians of Thailand	Family Medicine Residency	3 years	Diploma of the Thai Board of Family Medicine	Medical Proficiency Certificate (1 year): - Palliative care - Geriatric family medicine - Addiction family medicine Diploma of Thai Subspecialty Board of Palliative Medicine (2 years)
		Certificate of Completion of Training	5 years of clinical experience (achieve all the required entrustable professional activities and pass the examination)		
Vietnam (2001)	Ministry of Health Ministry of Education and Training	Family Medicine Residency	3 years	Diploma from the Ministry of Health	Specialist Degree 2 (diploma, 2 years)
		Master of Family Medicine	2 years	Master’s degree from the Ministry of Education and Training	
		Specialist Degree 1	2 years	Diploma from the Ministry of Health	

* Initially named as Primary Care Doctor (2017) and then transformed into Family Medicine Primary Care Specialist (2020).

**Challenges and future trends of postgraduate family medicine training**

Postgraduate family medicine training in Southeast Asia varies depending on the specific health concerns in each country. While family medicine faces both common and diverse challenges across the region, NCDs and the ageing population are major health concerns in several countries; in addition, the shortage of family physicians is also a significant challenge. The future trends of family medicine, country report cards and recent progress of each country are presented in Table 2.

**Table 2:** Challeng es and future trends of postgraduate family medicine training in Southeast Asia.

Country	Health concerns	Challenges of family medicine	Future trends of family medicine	Country report cards and recent progress
Indonesia	Triple burden of malnutrition (undernutrition, overweight and micronutrient deficiencies) NCDs Chronic infectious diseases (e.g. tuberculosis and HIV)	One family physician in every public health centre (*Puskesmas*) within 5 years among about 10,000 Puskesmas has been aimed. Hence, there is a need for recognising the prior competency (RKL) programme. Health equality has been addressed as an important challenge, especially in isolated, remote and frontier areas.	Workplace-based training and hospital-based training	The workplace-based programme enables trainees from all over Indonesia to take the programme while working in their respective healthcare facilities under supervision and immediately implementing what they have learnt to improve their practice.
Laos	Community health services at district hospital levels Ageing population Palliative care	One family physician in each district hospital across the country has been expected.	Family medicine provides primary care at university hospital and provincial levels.	Family medicine graduates, who work in district hospitals, are competent as 5-star doctors; they are responsible for both primary healthcare and primary care.
Malaysia	NCDs	Malaysia aims to have 8000 family physicians (1 family physician: 4000 population) to provide equitable healthcare for its growing population of over 30 million by 2050. Currently, there are around 1300 family physicians in Malaysia; the majority work in government health clinics.^9^	Unification of the training programmes through the launch of the National Postgraduate Medical Curriculum by the Medical Deans Council in August 2021. The National Postgraduate Medical Curriculum is a collaborative effort between universities, the Ministry of Health and the Academy of Medicine of Malaysia (representing medical professional societies and the private sector). The National Postgraduate Medical Curriculum has clearly outlined similar entry and exit criteria, structured supervised specialist training and core competencies required on completion of training for both the master’s programmes and parallel pathway in a unified and structured manner.^10^	The master’s programmes in family medicine produce around 100-150 family physicians per year, while about 200 family physicians are produced per year via the two existing parallel pathways. In the Malaysia Health White Paper 2023, there is a focus on transforming primary healthcare, and primary healthcare providers will be an important first point of contact for the population.^11^
Myanmar	Double burden of communicable diseases and NCDs due to poverty and political instability^12^	Graduates of the Diploma in Family Medicine have not been recognised as family physicians/specialist physicians. Due to the COVID-19 pandemic and political unrest, the Master’s Degree in Family Medicine programme has not started, and the Diploma in Family Medicine programme has been suspended since 2021.	The Diploma in Family Medicine will be resumed at the University of Medicine, Yangon, Myanmar, in the next academic year, 2025.	The Postgraduate Diploma in Family Medicine has been issued to physicians since 2017.^13^
Philippines	NCDs Impact of urbanisation and climate change	The mandate of increasing the number of trained family physicians serving as frontline workers in primary care and community-based urgent care services served as a double edged-sword for UHC. The increasing number of residency training programmes has caused an inadequate number of qualified trainers and preceptors. This is confounded by the shift of clinical placements from hospital-centric clinics to more primary care- and community-based clinical experience.	Family medicine has been engaged to capacitate primary care physicians through residency training and contribute to the reorientation of basic medical education to primary care and public health.	UHC was passed into law in 2019. The law recognises the role of primary care physicians as the backbone of the health system. As such, the Philippine Academy of Family Physicians: a) has developed and implemented a National Primary Care training programme for the Doctors-to-the-Barrios programme of the Department of Health, through the innovative practice-based framework for primary care practice-ready training.^14^ b) has been in continuous partnership with the Department of Health in training family physicians for UHC by establishing training programmes in all Department of Health hospitals nationwide.^15^
Singapore	Ageing population NCDs	With the launch of the national healthcare transformation Healthier SG in 2023, there has been a shift of healthcare towards primary care in Singapore. This has resulted in a drastic increase in demand for primary care physicians, with an urgent need to build capacity in family medicine, including both family physicians and family medicine trainers. With the ageing population and multimorbidities, the already heavy family medicine training structure and curriculum need to include the management of more complex patients traditionally seen at hospitals and working with community care colleagues to improve the integration of health and social care.	The immediate and urgent need is to build capacity in family medicine, not only in increasing numbers and clinical competencies but also in nurturing family medicine educators and clinician scientists (researchers).	Since the inception of the respective training programmes, Singapore has trained 1767 Graduate Diploma in Family Medicine physicians, 958 Master of Medicine (Family Medicine) physicians and 254 FCFPS (fellowship) qualified family physicians (as of March 2024).^16^
Thailand	NCDs Ageing population	Thailand is expected to have at least 6500 family physicians in 2026. Based on the national family medicine database, 3732 doctors are members of the Royal College of Family Physicians of Thailand,^17^ while less than 300 family physicians per year have been certified since 2015.^18^	Since 2019, Certificates of Medical Proficiency (i.e. palliative care, geriatric family medicine and addiction family medicine) under the Royal College of Family Physicians of Thailand have been approved by the Medical Council of Thailand. Family medicine with special interests has been discussed as a trend of continuing medical education and continuing professional development.	Thailand’s Constitution of 2017 emphasises the importance of family physicians in the health system, establishing a primary healthcare system in which there are family physicians to care for the people in an appropriate proportion.^19^
Vietnam	Enhancing the capacity of primary healthcare systems through the family physician model NCDs Ageing population Access to care Pandemics and public health crises	Unclear job positions for family physicians Regulations on health insurance reimbursement for healthcare services provided by family physicians Training challenges: limited training programmes, lack of standardisation, insufficient trainers and lack of access to structured continuing education Systemic barriers: healthcare system fragmentation, slow-moving family medicine promotion policies and relatively low salary compared to other specialists Community perception: low awareness of the roles of family medicine	Developing family medicine specialist training for district-level physicians Training preventive medicine physicians to become family physicians serving the primary healthcare level, especially in rural areas Collaborating with international institutions to expand the training programmes Integrating family medicine into UHC	More than 2000 family medicine physicians have been trained through diverse training programmes and levels: family medicine residency, PhD and first- and second-level specialisations. Training programmes have partnered with international collaborators to improve the quality of training.

COVID-19: coronavirus disease 2019, HIV: human immunodeficiency virus, NCD: non-communicable disease, UHC: universal health coverage

## Discussion and conclusion

Formal postgraduate training in family medicine was officially established in the Philippines in 1972. However, the exact time point at which the discipline of family medicine was introduced in this region remains unclear. The term ‘family medicine’ is commonly used to describe medical specialists in family medicine and primary care across the eight countries. Four Southeast Asian countries - Brunei, Cambodia, Laos and Timor-Leste - are not members of WONCA Asia Pacific.^20^ However, information on family medicine training in Laos has been gathered through the authors’ networks (OP and KP). A previous publication reported the existence of postgraduate family medicine training in Cambodia.^4^ Additionally, on the website of Mercy Medical Center Cambodia, International Postgraduate Diploma in Family Medicine, which is a 3-year full-time programme launched in 2020, is offered.^21^ This diploma programme was developed in partnership with the Christian Medical College of Vellore in India and is delivered online. It is endorsed by the International Christian Medical and Dental Association and accredited by Loma Linda University in the United States.^21^

Family medicine training is at different stages of development across the region, and the curricula are influenced by the specific health concerns of each country. The shortage of family physicians is also recognised as a challenge in Southeast Asia. Various strategies have been implemented to address context-specific issues. To enhance family medicine training programmes, most authors emphasise the need to strengthen collaborations between stakeholders in medical education and other sectors, including policymakers and frontline providers, to optimise the resources within countries and the region. At the regional level, shared experiences and lessons learnt across Southeast Asian countries may inform the development of family medicine training programmes.

This article has two limitations. First, information on postgraduate family medicine training from Brunei, Cambodia and Timor-Leste was not included. The authors attempted to identify the postgraduate family medicine training programmes and key opinion leaders in these three countries through contacts, internet searches and meetings; however, limited information was available. Second, the information on health concerns, challenges, future trends and success stories was drawn from country-specific documents published in different languages and the perspectives of the country representative(s) in the author list of this article. This may introduce some biases. Therefore, such information should not be interpreted as official national policies or action plans, nor should it be used for direct comparisons between countries.

In conclusion, postgraduate family medicine training in Southeast Asia has advanced significantly in some countries, with well-established structures, curricula and delivery methods. Some have expanded beyond family medicine to include specific discipline-based training (subspecialisation) based on the healthcare needs of the country. However, information about family medicine and its training in Brunei, Cambodia and Timor Leste is scarce. As Southeast Asian countries face some common healthcare challenges, there are opportunities to strengthen collaboration in family medicine training. Establishing regional platforms for knowledge-sharing, curriculum development and capacity building could help address training gaps and enhance the quality of education. A call to action is necessary among medical educators, policymakers and professional organisations across Southeast Asia to form a dedicated coalition to systematically enhance family medicine education quality during regular regional conferences, devise faculty exchange programmes and share digital learning resources. While a unified accreditation system may not yet be feasible, fostering regional partnerships could promote collaboration among the family medicine fraternity in Southeast Asia.
